# The illusion of malignancy: a rare case of post-hysterectomy gossypiboma misinterpreted as ovarian cancer

**DOI:** 10.1097/RC9.0000000000000280

**Published:** 2026-02-12

**Authors:** Somayeh Hajiahmadi, Maryam Nazemi, Mohammadreza Elhaie

**Affiliations:** School of Medicine, Isfahan University of Medical Sciences, Isfahan, Iran

**Keywords:** diagnostic errors, foreign bodies, gossypiboma, hysterectomy, ovarian neoplasms, radiology

## Abstract

**Introduction and importance:**

Gossypiboma, or retained surgical sponge, is a rare iatrogenic complication post-surgery, often mimicking malignancies like ovarian cancer in postmenopausal women. This leads to unnecessary oncologic interventions, patient anxiety, and procedural risks. Incidence ranges from 1:1000 to 1:18 000 procedures, with hysterectomies at higher risk due to human errors in counting. Recognizing imaging features is crucial to prevent misdiagnosis, highlighting the importance of multidisciplinary evaluation and preventive protocols in gynecological surgery.

**Case presentation:**

A 56-year-old asymptomatic postmenopausal woman, 9 years post-hysterectomy for fibroids, underwent routine transabdominal ultrasound revealing a 7.23 × 15.63 × 16.30 cm heterogeneous right adnexal mass initially interpreted as malignant ovarian neoplasm by a junior radiologist. Senior review and CT scan showed whorled structure with sterile gas, suggesting gossypiboma.

**Clinical discussion:**

Gossypibomas can remain latent for years, presenting as fibrotic masses with gas on imaging, mimicking tumors. Diagnostic pitfalls arise from inexperience, as seen here, but pathognomonic signs like spoke wheel patterns aid differentiation. Complications include obstruction or fistula; management involves surgical removal. Prevention requires standardized counting, radiopaque markers, and education to eliminate never events.

**Conclusion:**

This case emphasizes the need for high suspicion of gossypiboma in post-hysterectomy adnexal masses to avoid erroneous cancer diagnoses. Adhering to safety protocols enhances surgical integrity and patient outcomes.

## Introduction

Ovarian masses detected in postmenopausal women are frequently regarded with a high index of suspicion for malignancy, given the elevated risk in this demographic^[1–3]^. This concern often leads to expedited referrals to oncology specialists and potential interventions ranging from surgical resection to adjuvant therapies. However, not all such masses are neoplastic; benign mimics, including iatrogenic complications from prior surgeries, can present with similar imaging characteristics. One such entity is the retained surgical foreign body, commonly termed gossypiboma (derived from the Latin gossypium for cotton and Swahili boma for place of concealment), which refers to inadvertently retained textiles like surgical sponges or gauze during operative procedures^[4]^.

Gossypibomas represent a rare but preventable surgical complication, with reported incidences varying widely depending on the procedure and healthcare setting. Estimates suggest an overall rate of retained surgical items ranging from 1 in 5500 to 1 in 18 000 operations globally, while for abdominal surgeries specifically, the incidence may be as high as 1 in 1000 to 1 in 1500 procedures. Surgical sponges account for the majority (approximately 50%–70%) of these retained items, often due to human factors such as intraoperative distractions, emergency procedures, or inadequate counting protocols. In gynecological contexts, particularly following hysterectomies, gossypibomas are disproportionately common in the abdomen or pelvis (around 50% of cases), with vaginal procedures also implicated in a subset of incidents. These retained materials can remain asymptomatic for extended periods – sometimes years or even decades – before manifesting as incidental findings on imaging or through complications such as chronic pain, infection, fistula formation, or bowel obstruction^[5,6]^.

**HIGHLIGHTS**

- Rare case of **post-hysterectomy gossypiboma** mimicking **ovarian malignancy.**
- **Asymptomatic presentation** discovered incidentally on routine ultrasound.
- **Initial misdiagnosis** by junior radiologist corrected through expert re-evaluation.
- **Characteristic imaging features** – whorled internal pattern and sterile gas pockets – key to diagnosis.
- **Surgical removal and histopathology** confirmed retained gauze with chronic foreign-body reaction.
- Emphasizes **importance of radiologic expertise and multimodal imaging** in avoiding unnecessary oncologic surgery.
- Underscores **need for strict surgical protocols and sponge-tracking systems** to prevent such “never events.”

On imaging, gossypibomas may mimic malignant ovarian masses due to their heterogeneous appearance, including fibrotic encapsulation, calcification, and internal gas collections from sterile air trapping or low grade bacterial colonization without overt sepsis. Classic radiological signs include the spoke-wheel pattern on ultrasound or CT, representing the whorled texture of the sponge fibers, though these may be subtle and overlooked by less experienced interpreters. Numerous case reports highlight this diagnostic pitfall in post-hysterectomy patients, where retained gauze has migrated transvaginally, intravesically, or into adjacent viscera, leading to misdiagnoses as ovarian cysts, tumors, or even acute appendicitis. Such errors can result in unnecessary oncologic workups, patient anxiety, and exposure to procedural risks^[7–11]^.

This case report details a 56-year-old woman with a history of hysterectomy 9 years prior, who was initially misdiagnosed with a malignant ovarian mass on ultrasound by a junior radiologist, only for subsequent evaluation to reveal a gossypiboma with sterile gas collection.

## Case presentation

A 56-year-old postmenopausal woman presented to the radiology department for a routine pelvic ultrasound as part of her annual health screening. She had no specific complaints at the time of evaluation, denying any abdominal pain, bloating, vaginal bleeding, changes in bowel or bladder habits, weight loss, or other symptoms indicative of pelvic pathology. Her medical history was notable for a total abdominal hysterectomy performed 9 years earlier for symptomatic uterine fibroids, which was reportedly uncomplicated at the time. She had no history of other abdominal surgeries, malignancies, or chronic illnesses. Social history included being a non-smoker with no alcohol or illicit drug use. Family history was unremarkable for hereditary cancers or gynecological disorders. She was not on any regular medications and had no known allergies.

On physical examination, the patient was afebrile with stable vital signs (blood pressure 118/76 mmHg, heart rate 78 beats per minute, and respiratory rate 16 breaths per minute). Abdominal examination revealed a well-healed midline surgical scar from her prior hysterectomy, with no tenderness, masses, or organomegaly palpable. Pelvic examination was normal, showing no adnexal tenderness or masses on bimanual palpation. The remainder of the systemic examination was unremarkable.

The initial transabdominal ultrasound was performed by a radiology resident with limited experience in gynecological imaging. The scan demonstrated a heterogeneous, irregularly shaped mass measuring approximately 17.23 × 15.63 × 16.30 cm in the right adnexal region, adjacent to the ovarian remnant or pelvic sidewall. The mass exhibited mixed echogenicity with hypoechoic and hyperechoic components, ill-defined borders, and minimal vascularity on color Doppler, raising suspicion for a malignant ovarian neoplasm, such as an epithelial ovarian carcinoma. No free fluid or other abnormalities were noted in the pelvis. Based on these findings, the patient was urgently referred to a gynecologic oncology surgeon for evaluation and potential surgical intervention.

The oncology surgeon, upon reviewing the history and initial imaging report, noted the patient’s asymptomatic status and the remote history of hysterectomy, which prompted a request for a second opinion from a senior radiologist with over 20 years of experience in pelvic imaging. A repeat transabdominal ultrasound was conducted by the experienced radiologist, who reinterpreted the mass as having features inconsistent with a primary ovarian malignancy. Specifically, the mass showed a whorled, layered internal structure with central hyperechoic areas suggestive of trapped gas or foreign material, rather than solid tumor nodularity or cystic septations. The borders appeared more encapsulated than infiltrative, and there was no associated ascites or lymphadenopathy. These characteristics raised suspicion for a retained surgical foreign body, such as a gossypiboma, from the prior hysterectomy.

To further characterize the lesion and rule out complications like infection or malignancy, a contrast-enhanced computed tomography (CT) scan of the abdomen and pelvis was ordered. The CT revealed a well-circumscribed, ovoid mass in the right pelvic cavity with a density of approximately 20–30 Hounsfield units peripherally (consistent with fibrotic encapsulation) and central low attenuation areas indicative of sterile gas collections. The mass measured approximately 17 cm in maximal dimension, consistent with the transabdominal ultrasound measurements. No rim enhancement, surrounding inflammatory changes, or solid components suggestive of tumor were observed. The gas pockets were non-dependent and lacked air fluid levels, supporting a diagnosis of trapped sterile air within a retained foreign body rather than an abscess or necrotic tumor. Coronal and sagittal reconstructions confirmed the mass’s proximity to the hysterectomy bed, with no involvement of adjacent structures such as the bladder, rectum, or ureters.

Differential diagnoses at this stage included gossypiboma (retained surgical sponge), ovarian remnant syndrome with cystic degeneration, pelvic abscess, or a benign adnexal cyst. However, the imaging features strongly favored a retained foreign body given the history and absence of inflammatory markers (white blood cell count 6.2 × 10^9^/L and C-reactive protein <5 mg/L).

The patient was counseled regarding the findings and consented to exploratory laparotomy for definitive diagnosis and removal. Intraoperative exploration via a Pfannenstiel incision revealed a firm, encapsulated mass adherent to the pelvic sidewall. Upon dissection, retained surgical gauze from the prior hysterectomy was identified, surrounded by fibrous tissue and containing pockets of sterile gas without purulence or odor. The foreign body was completely excised, and the pelvic cavity was irrigated.

Postoperatively, the patient recovered without complications. She was discharged on postoperative day 3 with prophylactic antibiotics and analgesics. At a 2-week follow-up visit, she remained asymptomatic with a well healing incision. Long-term follow-up at 6 months confirmed no recurrence of symptoms or imaging abnormalities.

This case highlights the diagnostic journey from initial misinterpretation to accurate identification through escalating expertise and imaging modalities, ultimately preventing an unwarranted oncologic procedure.

## Discussion

Gossypiboma, alternatively termed textiloma or retained surgical sponge, constitutes a rare yet preventable iatrogenic complication resulting from the inadvertent retention of surgical textiles, such as sponges or gauze, within the operative site. Incidence estimates vary considerably, ranging from 1 in 1000 to 1 in 18 000 across all surgical interventions, with abdominal and gynecological procedures – particularly hysterectomies – exhibiting elevated risks, comprising up to 50%–70% of documented cases. Predisposing factors encompass emergent surgeries, elevated body mass index, procedural modifications intraoperatively, and lapses in sponge accounting protocols, rather than inherent surgical complexity^[1,11–17]^.

The index case involves a 56-year-old postmenopausal woman with a remote history of hysterectomy, initially misdiagnosed with an ovarian malignancy via ultrasound by a novice radiologist, thereby exemplifying the diagnostic challenges inherent to gossypibomas. The long delay – spanning 9 years in this instance – aligns with literature indicating that such entities may remain indolent for extended durations, manifesting incidentally or through secondary complications. The patient’s asymptomatic profile is congruent with reports wherein gossypibomas undergo fibrotic encapsulation, presenting as imaging aberrations that simulate neoplastic processes. In postmenopausal cohorts, adnexal masses elicit heightened oncologic vigilance, predisposing to erroneous referrals and interventions, as evidenced herein^[4,12–14,18–20]^. As summarized in Table 1, similar cases in the literature demonstrate varied latencies and diagnostic challenges.

**Table 1:** Summary of selected gossypiboma cases mimicking ovarian tumors

Author/year	Case description (latency, symptoms)	Diagnostic process (imaging used, initial misdiagnosis)	Outcome	Key lesson
Zhang *et al* (2017)	Post-cesarean section; latency not specified; presented as abdominal cystic lump.	CT showed whorl like appearance with gas bubbles; initially misdiagnosed as ovarian teratoma.	Surgical resection with adhesive intestine loop; confirmed gossypiboma.	Always consider gossypiboma for cystic masses in patients with surgical history to avoid misdiagnosis.
Noei *et al* (2021)	41-year-old post-laparotomy for ovarian adenocarcinoma; 3-year latency; asymptomatic, incidental mass.	Imaging (ultrasound/CT) showed mass; misdiagnosed as recurrent ovarian tumor.	Laparotomy excision; no complications.	Asymptomatic gossypiboma should be suspected in post-laparotomy patients with masses.
Cengiz *et al* (2014)	Post-gynecologic procedure; 13-year latency; presented as ovarian mass.	Imaging suggested ovarian tumor; misdiagnosed as ovarian neoplasm.	Surgical removal; uneventful recovery.	Long latency periods possible; high suspicion needed for chronic masses post-surgery.
Rafat *et al* (2015)	22-year old post-caesarean; 2-year latency; abdominal mass.	Radiological imaging; misdiagnosed as ovarian dermoid cyst.	Laparotomy revealed and removed gossypiboma.	Gossypiboma can mimic benign ovarian cysts like dermoids, leading to unnecessary procedures.
Chopra *et al* (2015)	Series of eight cases, mostly post-gynecologic; varied latency (months to years); symptoms included masses, obstruction.	Preoperative diagnosis in 7/8 via imaging; one misdiagnosed as adnexal mass.	Surgical removal (laparotomy in most); good outcomes.	Varied presentations post-gynecologic surgery; strict counting protocols essential to prevent.
Varlas *et al* (2023)	28 year old post-emergency cesarean hysterectomy; 11-month latency; chronic lower abdominal pain, and palpable mass.	CT/MRI showed tumor like mass; misdiagnosed as neoplasm.	Laparotomy excision; no recurrence.	High risk in emergency cesareans; emphasizes need for checklists and team vigilance.

**Figure 1.**
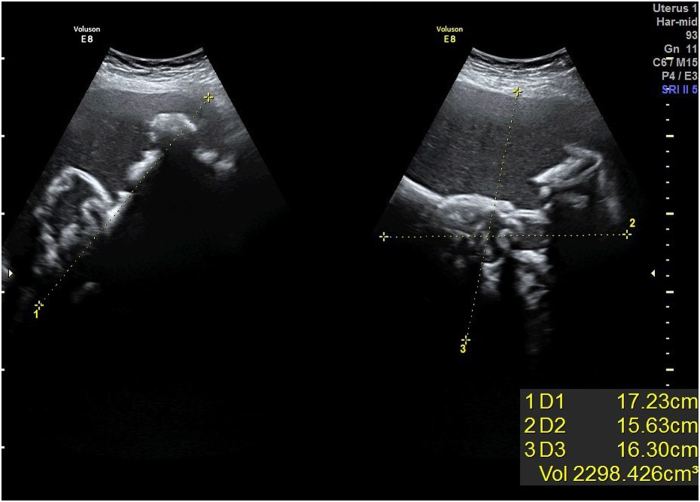
Transabdominal sonography of 56-year-old female reveals large cystic lesion with internal echo and linear echogenic regions with posterior acoustic shadow in dependent part.

**Figure 2.**
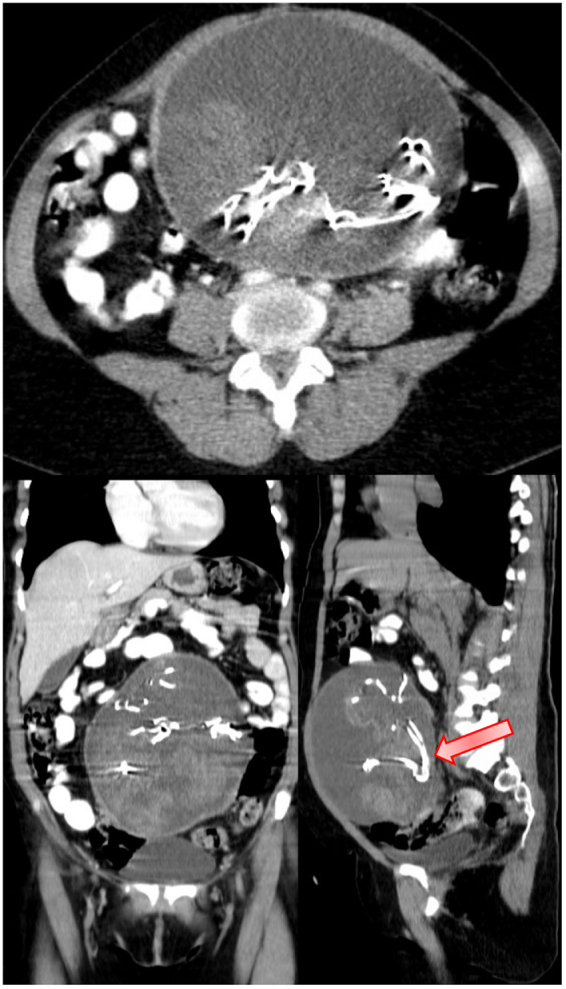
Contrast enhanced computed tomography (CT) of the abdomen and pelvis in a 56-year-old postmenopausal woman with a history of total abdominal hysterectomy.

Radiological acumen is paramount in adjudication, albeit susceptible to experiential disparities, as demonstrated by the initial misinterpretation. The preliminary transabdominal ultrasound delineated a heterogeneous mass with irregular margins and variegated echogenicity, interpreted as cancerous (Fig. 1A), yet reflective of gossypiboma’s characteristic hypoechoic periphery and hyperechoic core attributable to entrapped gas or calcific deposits. The pathognomonic spoke wheel or stratified configuration, discernible on ultrasound and CT, was ascertained solely upon senior radiologist review (Fig. 1B), emphasizing the imperative of specialized expertise in discerning nuanced attributes. CT corroborated sterile gas accumulations devoid of enhancement or inflammatory stigmata (Fig. 2A and 2B), distinguishing from abscesses or neoplasms, notwithstanding diagnostic hurdles posed by non-radiopaque materials. Analogous reports chronicle gossypibomas masquerading as ovarian dermoids, teratomas, or stromal neoplasms, with potential migration into contiguous viscera exacerbating interpretive complexity^[12,13,15,16]^.

Differentiating gossypiboma from ovarian tumors is essential to prevent diagnostic errors and unnecessary interventions. Gossypibomas are often asymptomatic for years or present with chronic, nonspecific symptoms such as mild abdominal discomfort or pain, whereas ovarian tumors typically cause more pronounced symptoms including bloating, early satiety, unintentional weight loss, acute pelvic pain, and changes in bowel or urinary habits. On clinical examination, gossypibomas manifest as firm, well-encapsulated masses without systemic involvement, in contrast to ovarian tumors which may feature ascites, palpable lymphadenopathy, or signs of cachexia. Imaging distinctions are key: gossypibomas display pathognomonic features like a spoke wheel or whorled pattern on ultrasound, with sterile gas collections and fibrotic encapsulation on CT without rim enhancement, while ovarian tumors appear as solid or complex cystic masses with septations, nodularity, increased vascularity on Doppler, and contrast enhancement. Blood tests often show normal inflammatory markers in gossypiboma cases, unlike potential elevations in CA-125 or other tumor markers for ovarian malignancies. Crucially, a history of prior abdominal surgery, such as hysterectomy, strongly favors gossypiboma over a primary ovarian neoplasm, as retained foreign bodies are iatrogenic complications. Integrating this surgical history with multimodal imaging and clinical findings ensures accurate differentiation and optimal patient care^[4,21,22]^.

**Figure 3.**
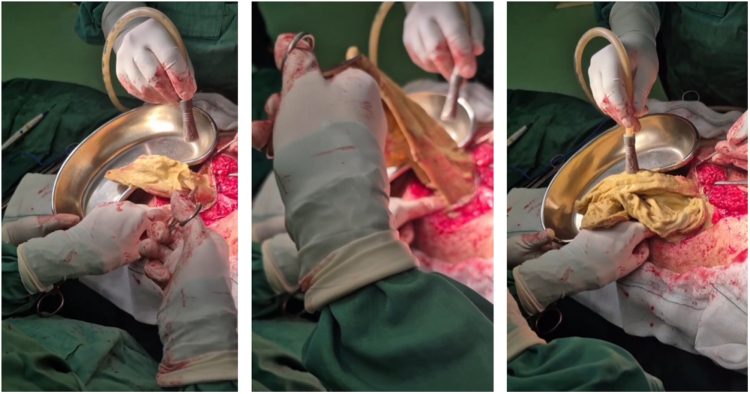
Intraoperative photographs demonstrating the surgical removal of a gossypiboma during exploratory laparotomy.

Complications span a spectrum from benign encapsulation to grave sequelae, including intestinal obstruction, fistulization, perforation, sepsis, or transmural translocation sans overt apertures. Herein, sterile gas, ostensibly derived from occluded air during fibrogenesis or subdued microbial activity, absent systemic infection, attenuated acute morbidity. Therapeutic intervention entails surgical extirpation, frequently via laparotomy owing to adhesions, as executed with favorable postoperative convalescence (Fig. 3)^[14,15]^. To investigate the underlying reasons for the retained sponge in this case, we reviewed local surgical routines in Iranian contexts, where manual counting lapses are a primary contributor to gossypibomas, especially in gynecological procedures. A 3-year retrospective evaluation of retained foreign bodies in Iran (2008–2011) found sponges as the most common item (73%), with gynecology wards affected in 34% of cases, often due to human errors like incorrect counts rather than procedural complexity^[23]^. Standard protocols, such as the surgical safety checklist from Isfahan University of Medical Sciences and similar resources from Tehran University of Medical Sciences, require verbal verification of surgical supplies, gauze, and needles by the nurse, surgeon, and anesthesiologist before patient departure from the operating room; however, without mandatory adjuncts like X-rays or radiopaque markers, these remain vulnerable to oversights compared to global standards^[23]^.

## Strengths and limitations

This case report’s strengths include its detailed documentation of imaging progression from initial ultrasound misinterpretation to confirmatory CT, which illustrates key diagnostic pitfalls and aids educational value. The multidisciplinary approach, involving senior radiologist review and surgical confirmation, effectively prevented unnecessary oncologic surgery, highlighting practical benefits in clinical decision making. Additionally, the report adheres strictly to the SCARE guidelines, ensuring methodological rigor and transparency. However, as a single case study, it lacks generalizability to broader populations, limiting its applicability beyond similar postmenopausal post-hysterectomy scenarios. Furthermore, the follow-up was restricted to 6 months, with reliance on retrospective data, and there may be underreporting bias due to the medicolegal sensitivities surrounding gossypibomas.

Prophylaxis is foundational, mandating rigorous adherence to standardized inventories for sponges and instruments, integration of radiopaque indicators, barcode methodologies, and adjunctive intraoperative radiography in vulnerable scenarios. Etiologic inquiries implicate emergent contexts and communicative deficiencies, advocating augmented education and technological enhancements to relegate these as never events. To enhance prevention, radiofrequency identification tagged sponges offer a reliable technology, with studies showing they can detect retained items in seconds and potentially prevent over 97% of cases by supplementing manual count. For high-risk procedures like hysterectomies, mandatory intraoperative X-rays are recommended when counts are unresolved or in emergencies, as they help identify radiopaque markers despite a sensitivity of about 67% for sponges. Team training programs, such as Team STEPPS, improve communication and reduce errors by addressing human factors like distractions^[15]^. The WHO Surgical Safety Checklist ensures standardized counts and briefings, leading to better compliance and lower morbidity rates. Institutional audits track deviations and evaluate protocols, fostering a culture of safety through regular reviews. Finally, hospitals should update policies to integrate these technologies and steps, making gossypibomas true never events through ongoing education and system improvements^[24–26]^.

The present narrative, consonant with extant cohorts, reinforces the exigency for interdisciplinary circumspection, consultative reevaluations, and multimodal diagnostics to forestall diagnostic fallacies and ameliorate prognoses in postsurgical demographics.

## Conclusion

This example of a gossypiboma erroneously adjudged as an ovarian malignancy 9 years post-hysterectomy shows the interpretive intricacies and latent iatrogenic perils attendant to retained operative artifacts, as vividly illustrated through sequential imaging (Figs. 1A, 1B, 2A, and 2B) and surgical confirmation (Fig. 3). It accentuates the indispensable synergy of radiologic proficiency, meticulous anamnesis, and sophisticated imaging in disentangling enigmas, thereby obviating superfluous oncologic escalations and concomitant psychosocial burdens. Practitioners are enjoined to sustain elevated suspicion for gossypibomas in individuals with antecedent abdominal interventions, notably hysterectomies, wherein prevalence is amplified. In summation, stringent conformance to prophylactic paradigms, encompassing systematized accounting and innovative modalities, is indispensable to extirpate these avertible adversities and fortify operative integrity.

## Data Availability

The data that support the findings of this study are available from the corresponding author upon reasonable request.
